# Supplementary material for: Taxonomic revision of the tarantula genus Aphonopelma Pocock, 1901 (Araneae, Mygalomorphae, Theraphosidae) within the United States
Source: Zookeys. 2016 Feb 4;(560):1–340. doi: 10.3897/zookeys.560.6264 (PMC4768370; doi:10.3897/zookeys.560.6264)

Tarsus 1/Metatarsus 3 Length ratio − females

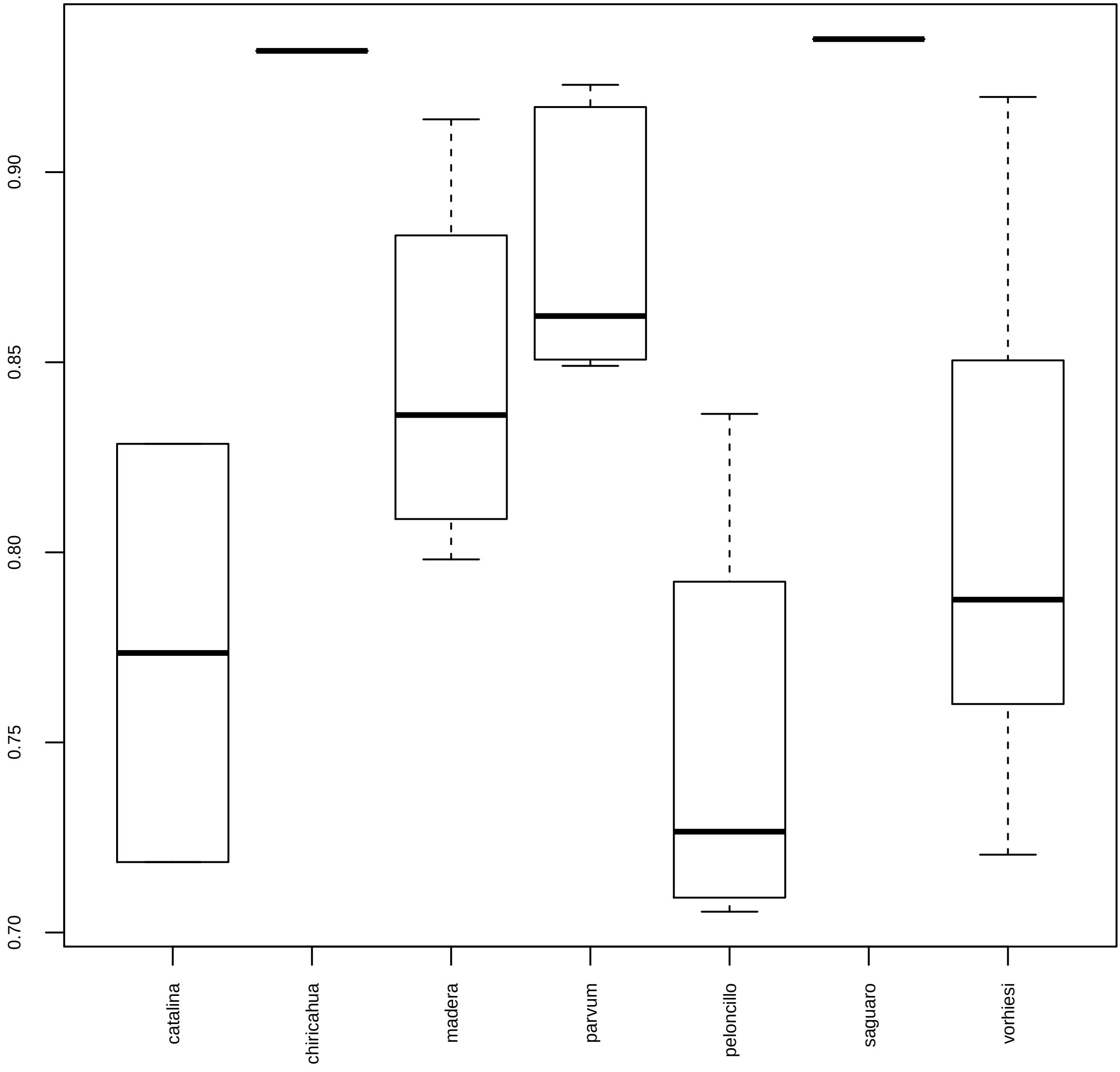

Supplement: Supplementary material 2 — Compressed files (.zip) containing Aphonopelma boxplot and PCA morphospace data. [file zookeys-560-001-s002.zip › Supp2b.zip!/Appendix 2B/Boxplot_morphospace/Aphonopelma_boxplots_females/Sky Islands/A1M3_ratio_females.pdf]
